# Effects of laterally wedged insoles on static balance in patients with medial compartment knee osteoarthritis

**DOI:** 10.1186/1757-1146-7-S1-A22

**Published:** 2014-04-08

**Authors:** Fariba Ahmadi, Saeed Forghany, Christopher Nester, Richard Jones

**Affiliations:** 1 Musculoskeletal Research Centre, Isfahan University of Medical Sciences, Iran; 2 Centre for Health Sciences Research, University of Salford, UK

## Background

Patients with knee OA usually present with major involvement in the medial compartment characterized by joint inflammation, loss of cartilage and joint space and experience increased loads across this compartment. These contribute to pain, changes in muscle control and may interfere with balance and postural control [1,2].

Laterally wedged insoles or footwear components have been used in different forms to alter the knee adduction moments that are associated with knee pain and progression of knee OA. They have generally been shown to have immediate beneficial effects on knee loading, pain and physical performance during walking and stair climbing [3,4]. The effects on standing balance and posture control have not been reported, nor have different designs of lateral wedge components (insole vs. shoe sole modification) been compared. The aim of this study was to investigate the effects of four different designs of lateral wedges on static balance in patients with knee osteoarthritis.

## Methods

18 patients (age 59.6 ± 5.8 years) with painful knee OA confirmed by an orthopaedic surgeon were recruited. Static balance was assessed using a force plate (AMTI, 1000 Hz) during 60s double leg standing. Movement of the center-of-pressure (COP) was measured under five shod randomised conditions: (1) no wedge; (2) 8.5 ° lateral heel wedge (inside shoe); (3) 8.5 ° lateral heel and forefoot wedge (inside shoe); (4) 8.5 ° lateral heel wedge (shoe sole); (5) 8.5 ° lateral heel and forefoot wedge (shoe sole).

Balance control was quantified using the amplitude and velocity of centre of pressure (COP) data in the middle 20s. The results were statistically analyzed using the nonparametric Fridman test followed by Wilcoxon Signed Rank.

## Results

Whilst there was a trend for COP parameters to decrease when wearing of the various lateral wedges compared to no-wedge condition, differences did not reach significance (Table 1).

**Table 1:** Mean differences in COP parameters between different lateral wedges conditions and no- wedge condition

	Lateral Wedge conditions			
	**8.5 ° Heel wedge (Insole)**	**8.5 ° Heel & Forefoot wedge (Insole)**	**8.5 ° Heel wedge (shoe sole)**	**8.5 ° Heel & forefoot wedge (shoe sole)**
**Total Mean distance (mm)**	-0.036	-0.83	-0.98	-1.21
**Mean COP velocity(mm/sec)**	5.89	-1.27	-1.19	-0.35
**95% confidence circle area (mm^2^)**	-48.09	-205.79	-249.25	-286.52
**95% confidence ellipse area (mm^2^)**	-30.04	-137.42	-219.95	-216.13

The total mean distance over the 20 seconds, the 95% confidence circle area and 95% confidence ellipse area were all statistically significantly greater when wearing the in-shoe lateral heel wedge compared to all shoe sole wedge conditions (Table 2).

**Table 2:** Mean differences of COP parameters between different the heel wedge insole and the various out-shoe lateral wedges conditions

	lateral heel wedge insole versus:	Mean differences	P^*^
**Total Mean distance (mm)**	8.5 ° Heel Wedge (shoe sole)	-0.95	0.02
	8.5 ° Heel & Forefoot wedge (shoe sole)	-1.17	0.03
**95% confidence circle area (mm^2^)**	8.5 ° Heel & Forefoot wedge (shoe sole)	-238.43	0.04
**95% confidence ellipse area (mm^2^)**	8.5 ° Heel Wedge (shoe sole)	-189.91	0.04
	8.5 ° Heel & Forefoot wedge (shoe sole)	-186.08	0.006

## Conclusion

Balance was not affected by any of the shoe sole lateral wedges, but COP excursion increased when wearing insole lateral heel wedges, suggesting a deterioration in standing balance. Changes in plantar loading, ankle moments and foot position due to the wedge, and shoe fit, may account for this change in standing balance.
